# Rapid Microfluidic Ion-Exchange Optode System for Point-of-Care Determination of Sodium Concentration in Serum

**DOI:** 10.3390/bios15020104

**Published:** 2025-02-12

**Authors:** Kuan-Hsun Huang, Cheng-Xue Yu, Chia-Chun Lee, Chin-Chung Tseng, Lung-Ming Fu

**Affiliations:** 1 Department of Engineering Science, National Cheng Kung University, Tainan 701, Taiwan; n96104496@gs.ncku.edu.tw (K.-H.H.); n98121509@gs.ncku.edu.tw (C.-X.Y.); 2 Division of Nephrology, Department of Internal Medicine, National Cheng Kung University Hospital, College of Medicine, National Cheng Kung University, Tainan 704, Taiwan; n045740@mail.hosp.ncku.edu.tw (C.-C.L.); chinchun@mail.ncku.edu.tw (C.-C.T.); 3 Institute of Clinical Medicine, College of Medicine, National Cheng Kung University, Tainan 704, Taiwan

**Keywords:** sodium, microfluidic, finger pump, micro-spectrometer, chronic kidney disease

## Abstract

A microfluidic system for detecting sodium ions (Na^+^) has been developed, incorporating a micro finger-pump chip and a micro-spectrometer platform to measure Na^+^ concentration in human serum. A small volume (10 μL) of serum sample is introduced into the microchip and reacted with a preloaded reagent mixture through a two-step finger-pump actuation process. The resulting purple complex is directed into the detection area of the chip and analyzed using the micro-spectrometer at wavelengths of 555 and 666 nm. The Na^+^ concentration is then inversely derived from the measured A_555_/A_666_ absorbance ratio using self-written software installed on a Raspberry Pi. The entire detection process is completed in just 3 min, offering a significant advantage in meeting clinical needs compared to the traditional reporting turnaround time of several hours in medical institutions. The experimental results indicate a linear relationship between the measured absorbance ratio and Na^+^ concentration within the range of 1–200 mM, with a correlation coefficient of R^2^ = 0.9989. Additionally, the detection results from 60 serum samples collected from chronic kidney disease (CKD) patients showed a strong agreement with those obtained using the conventional indirect ion-selective electrode (ISE) method, achieving a correlation coefficient of R^2^ = 0.9885 and an average recovery rate of 99.4%. In summary, the proposed system provides a practical, affordable, and rapid alternative to conventional Na^+^ detection methods, making it highly promising for point-of-care (POC) testing applications.

## 1. Introduction

Na^+^ account for approximately 90% of the total cations in human blood serum and are thus highly representative and clinically significant in medical diagnostics. As the primary extracellular cation in the body, Na^+^ play a crucial role in many physiological processes, including maintaining extracellular fluid osmotic balance, regulating muscle contraction, maintaining acid-base balance, and assisting nerve conduction [1,2]. In normal healthy adults, the serum Na^+^ concentration ranges from 135 to 145 mM. A Na^+^ concentration below 135 mM indicates hyponatremia, with clinical manifestations such as nausea, vomiting, muscle cramps, and even seizures. Conversely, when the Na^+^ concentration exceeds 145 mM, hypernatremia occurs, which can lead to symptoms such as thirst, fatigue, muscle weakness, and central nervous system abnormalities [3,4]. Na^+^ balance, both hyponatremia and hypernatremia, is a common issue in chronic kidney disease (CKD) patients. Thus, regular Na^+^ monitoring is essential for detecting and monitoring CKD.

Many methods are available for quantifying the concentration of Na^+^ in serum, including ion-selective electrodes [5,6,7], spectroscopy detection [8], fluorescence detection [9,10,11], and electrochemical detection [12,13,14,15,16]. While these methods are widely used and offer accurate results, certain configurations may involve the use of expensive precision instruments or complex operational procedures. Consequently, the development of cheaper and more straightforward point-of-care testing (POCT) systems [17,18,19,20] has emerged as an important concern.

Microfluidic technology has attracted significant interest in recent years owing to its low sample and reagent consumption, fast throughput, high sensitivity, compact size, and relatively economy [21,22,23,24,25,26]. It has found extensive applications in many fields, including medicine [27,28,29], biosensors [30,31,32,33], food safety [34,35,36,37,38], chemical engineering [39,40,41], and industrial manufacturing [42,43,44,45]. Lee et al. [46] developed an ion-selective optode sensor cartridge for the detection of potassium, Na^+^, and chloride ions in serum. The accuracy and selectivity of the proposed method were improved by integrating a dried buffer layer into the device, which automatically adjusted the pH. The detection results obtained for potassium, Na^+^, and chloride ions were in excellent agreement with the measurements acquired using a commercial system (cobas^®^ 8000 modular analyzer, Roche Diagnostics, Basel, Switzerland), achieving correlation coefficients of 0.976, 0.955, and 0.966, respectively.

This study presents a microfluidic Na^+^ ion-exchange optode system for measuring Na^+^ concentrations in human serum samples. The system consists of a finger-pump-operated microfluidic chip and a micro-spectrometer detection platform. Prior to the detection process, the required reagents are preloaded into the reagent chamber and sealed. The serum sample is then injected into the sample chamber, and finger pressure is applied to prompt the mixing of the reagents and sample in a serpentine channel, triggering a colorimetric reaction between them. After the reaction process, the chip is placed into the micro-spectrometer platform, where the absorbance ratio of the reaction complex is measured and used to calculate the Na^+^ concentration inversely. This study provides a more time-efficient and cost-effective detection method, offering a point-of-care testing (POCT) application and a more reliable approach for CKD detection.

## 2. Materials and Methods

### 2.1. Fabrication of Microfluidic Na^+^ Detection Chip

**Figure 1:**
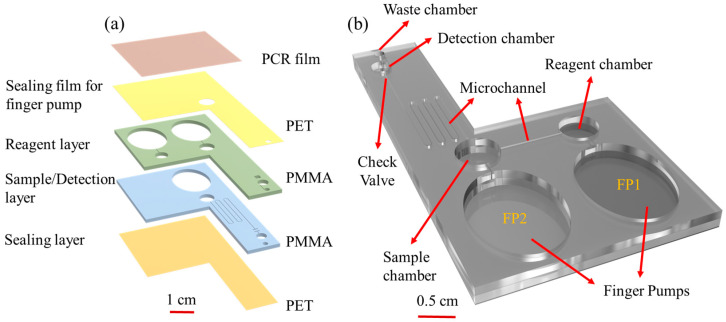
(**a**) Multilayer structure of chip; (**b**) schematic illustration of chip.

Figure 1a shows an exploded view of the proposed finger-pump microchip. The chip is composed of a PCR plate sealing film (MSB1001, Genmall Biotech. Co., Ltd., Tainan, Taiwan) and an upper PET (polyethylene terephthalate) layer (purchased from a local stationery store), both with a thickness of 0.1 mm. It also includes two PMMA (polymethyl methacrylate) substrates (purchased from a local stationery store), each 1.5 mm thick, which contain the finger pumps, reagent chamber, sample chamber, serpentine channel, detection chamber, and a check valve. The structure is sealed by a lower PET layer. A check valve is set up in front of the detection area, and PDMS (polydimethylsiloxane) is used as a material to block the backflow of the reagent to prevent the reagent from flowing back after entering the detection area.

Figure 1b shows the detailed arrangement of the reagent and sample detection layers. The two oval-shaped finger pumps have dimensions of 15 × 20 mm^2^, while the reagent chamber, serum sample chamber, and detection chamber have diameters of 7 mm, 4 mm, and 3 mm, respectively. The serpentine mixing channel has a total length of approximately 125 mm. The different sections of the chip are connected by microchannels, which have widths of 180 μm and depths of 90 μm, respectively. The chip has an overall size of 40 × 70 mm^2^.

The PET and PMMA layers were bonded through oxygen plasma treatment, while the PMMA layers were assembled using thermal compression bonding. The chambers and microchannels in the two PMMA chips were created using a CO_2_ laser system (Giant Technology Co., Ltd., Miaoli, Taiwan). An operating detailed description of the laser processing method can be found in [47].

### 2.2. Experimental Procedures and Smart Analysis Device

A volume of 100 μL of reagent solution was placed into the reagent chamber and sealed with a PET strip. Subsequently, 10 μL of serum sample was added to the sample chamber and sealed with a PCR plate sealing film. Finger pump 1 (FP1) was activated to drive the reagent into the sample/mixing chamber and initiate Na^+^ ion exchange. The second finger pump (FP2) was then used to drive the reaction solution through the serpentine channel to complete the mixing and reaction process. The finger pump was actuated several times until the reaction solution started to flow into the waste chamber, indicating that the detection chamber was filled. Finally, the chip was rotated through 90° and inserted into the chip holder of the detection system.

**Figure 2:**
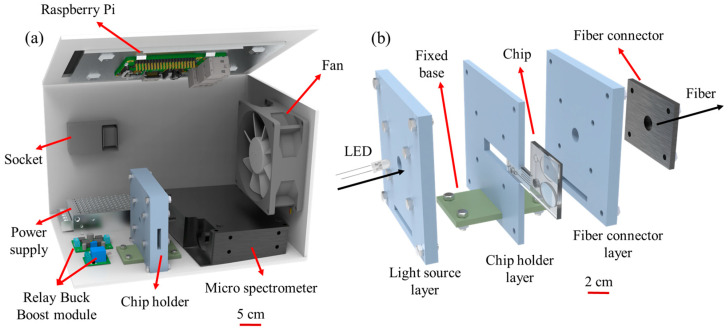
(**a**) Schematic illustration of detection system and (**b**) chip holder.

As illustrated in Figure 2a, the detection system comprised a buck/boost converter module (DC-DC, 4A-XL6009E1, XLSMIL, Shanghai, China), a micro-spectrometer (SE2020-025-VNIR, SmartEngine, Hsinchu, Taiwan), a 5V power supply (RS-15-5, Meanwell Co., New Taipei City, Taiwan), a relay block (KY-019, SONGLE, Taipei, Taiwan), a Raspberry Pi touch tablet computer (Raspberry Pi 3 Model B, Raspberry Pi Foundation, Tokyo, Japan), a chip holder, and a cooling fan (Axial Fan, RS Co., Ltd., New Taipei City, Taiwan). In the detection process, the detection chamber of the chip was illuminated by a white light LED, and the reaction complex was observed by the micro-spectrometer. The resulting optical signal was passed to the Raspberry Pi via an optical fiber and processed by self-written software to determine the Na^+^ ion concentration.

### 2.3. Preparation of Standard Na^+^ Solutions and Reagents

We added 12.1 g of Tris to approximately 800 mL of deionized (DI) water and stirred until completely dissolved. Concentrated HCl was added dropwise until the pH reached 7.4. The final volume was adjusted to 1 L by adding DI water, creating a 0.1 M Tris-HCl buffer at pH 7.4. To prepare a 2.14 M stock solution, 5 g of NaCl was dissolved in 40 mL of the Tris-HCl buffer. This stock solution was then serially diluted with Tris-HCl buffer to produce five NaCl standard samples with Na^+^ concentrations ranging from 1 to 200 mM.

For the reagents, 10 mg of chromoionophore I (ETH5294) was dissolved in 300 μL of THF to yield a final concentration of 19 mM. Additionally, 50 mg of potassium tetrakis [3,5-bis(trifluoromethyl)phenyl]borate (KTFPB) was dissolved in 1000 μL of THF, resulting in a final concentration of 39.5 mM. Finally, 100 mg of sodium ionophore VI was dissolved in 1000 μL of THF to obtain a final concentration of 150.9 mM. Table 1 shows the details of the sources of the reagents.

### 2.4. Collection of Real-World Serum Samples

Serum samples were collected from patients with CKD and healthy volunteers at National Cheng Kung University Hospital, a public teaching hospital in Taiwan. Whole blood samples were collected in vacutainer, allowed to stand at room temperature for 30 min, and then centrifuged at 3000× *g* for 15 min to separate the serum from the blood clot. The serum was transferred into sterile tubes using a pipette and stored at −80 °C until further use.

All participants provided fresh whole blood samples and written informed consent in accordance with the Institutional Review Board protocol of National Cheng Kung University Hospital (IRB-A-ER-108-527). Each sample was assigned a unique identification code based on the donor, the date of collection, and the collection method. No personal identifying information was recorded.

## 3. Results and Discussion

### 3.1. UV–Visible Spectrophotometry Detection of Na^+^

**Figure 3:**
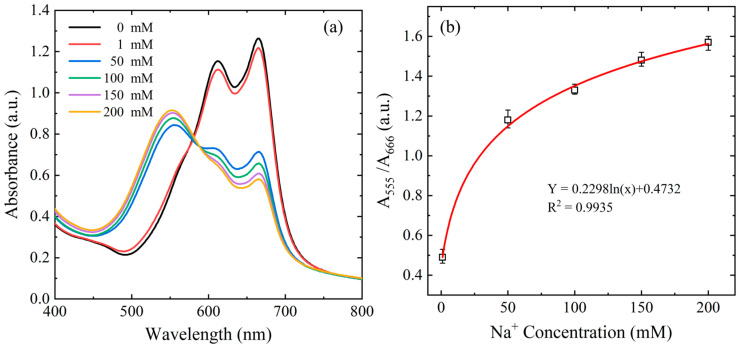
(**a**) Ultraviolet–visible spectrophotometer results for variation in absorbance value with sodium ion concentration in range of 1–200 ppm. (**b**) Calibration curve established from spectrophotometer results.

An amount of 100 μL of NaCl standard solutions with known concentrations in the range of 1–200 mM were added to mixtures consisting of 1000 μL of ETH5294, KTFPB, ionophore, and Tris-HCl buffer, and stirred for one min to perform ion exchange. The resulting solutions were analyzed using a UV–visible spectrophotometer (Jasco V-700, Tokyo Corporation, Tokyo, Japan). As the Na^+^ concentration increased, the solution color shifted from blue to purple. Additionally, as illustrated in Figure 3a, the peak intensity at 555 nm increased, while the peak at 666 nm decreased. Figure 3b displays the variation in the calculated absorbance ratio (A_555_/A_666_) as a function of Na^+^ concentration. A regression analysis showed a logarithmic relationship between the absorbance ratio (Y) and Na^+^ concentration (X), expressed as Y = 0.2298ln(X) + 0.4732, with a correlation coefficient (R^2^) of 0.9935. This indicates that the absorbance ratio reliably reflects the Na^+^ ion concentration within the 1–200 mM range.

### 3.2. Optimal Reaction Conditions for Na^+^ Concentration Detection

For the detection system proposed in the present study, the measured absorbance ratio (A_555_/A_666_), and hence the detection performance, depends on two key factors: (1) the concentrations of the different reagents, and (2) the detection path length.

#### 3.2.1. Reagent Concentration

**Figure 4:**
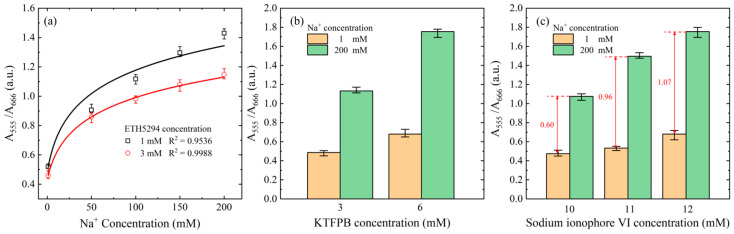
(**a**) Variation in different ETH 5249 concentrations with sodium ion concentration in the range of 1 mM to 200 mM. (**b**) Differences in absorbance ratios among different concentrations of KTFPB were investigated under sodium ion concentrations of 1 mM and 200 mM. (**c**) Differences in absorbance ratios of different ionophore concentrations to 1 mM and 200 mM sodium ion concentrations.

In general, using a few indicator results in an incomplete color change corresponding to the Na^+^ concentration, while too much indicator adversely affects the reagent’s coloration. To determine the optimal reagent concentration for the reaction, the absorbance ratios of five NaCl control samples were measured with ETH5294 indicator concentrations of 1 mM and 3 mM. For each sample and indicator solution, the absorbance ratio was measured using the micro-spectrometer in the proposed detection platform. As shown in Figure 4a, the absorbance ratio decreased as the indicator concentration increased. However, a higher ETH5294 concentration resulted in a significantly improved correlation coefficient (R^2^ = 0.9988). At lower concentrations, the amount of ETH5294 is not sufficient to bind to all released H ions, causing the solution color to not fully appear. Thus, 3 mM was selected as the optimal ETH5294 concentration for accurate Na^+^ detection in all the remaining experiments.

As an ion additive, KTFPB enhances ion mobility in the solution and improves the ion exchange efficiency [48]. Figure 4b shows the measured absorbance ratios for two of the NaCl control samples with concentrations of 1 mM and 200 mM, respectively, and KTFPB concentrations of 3 mM and 6 mM. As shown, a higher KTFPB concentration increased the absorbance ratio, particularly at higher Na^+^ concentrations. To enhance the detection sensitivity, it is desirable to increase the change in the absorbance ratio for different Na^+^ concentrations. Thus, a KTFPB concentration of 6 mM was selected as the optimal condition for the reaction process in all the following experiments.

Finally, adjust the sodium ionophore VI carrier concentration to ensure that when the carrier combines with sodium ions, it can release the number of H ions corresponding to the current sodium ion concentration to optimize the performance of the detection system and thereby promote color change. As shown in Figure 4c, a carrier concentration of 11 mM resulted in a greater increase in the absorbance ratio at different Na^+^ concentrations than a concentration of 10 mM. Although a carrier concentration of 12 mM further increased the difference between the absorbance ratios of the two samples, the enhancement effect (1.07) was not significantly higher than that for a concentration of 11 mM (0.96). Thus, for experimental cost and convenience considerations, the lower carrier concentration of 11 mM was chosen as the optimal concentration.

#### 3.2.2. Detection Path Length

**Figure 5:**
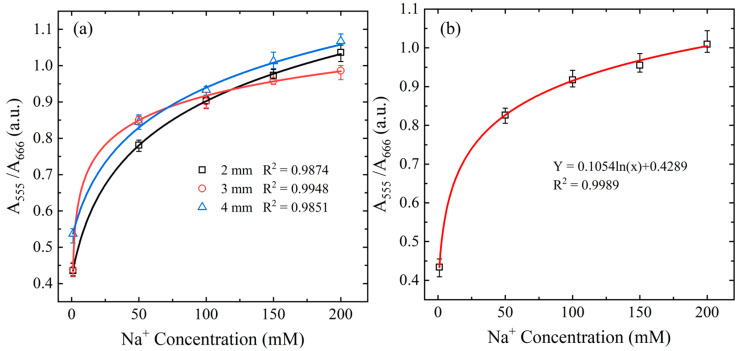
(**a**) Variation in absorbance ratio A_555_/A_666_ with Na^+^ concentration given different PMMA layer thicknesses. (**b**) Variation in absorbance ratio A_555_/A_666_ with Na^+^ concentration given optimal reaction conditions and chip thickness.

According to the Beer–Lambert law, under the same absorbing medium conditions, the absorbance value is directly proportional to the optical path length. Therefore, designing an appropriate optical path length (chip thickness) is essential to optimize the detection performance. Various optical path lengths were considered (2 mm, 3 mm, and 4 mm), corresponding to reagent solution volumes of 100 μL, 150 μL, and 200 μL, respectively. Figure 5a illustrates the change in the A_555_/A_666_ absorbance ratio as a function of Na^+^ concentration for each optical path length. As shown, the chip with a total PMMA layer thickness of 3 mm yielded the highest correlation coefficient (R^2^ = 0.9948) and was thus selected as the optimal PMMA layer thickness in the chip design.

#### 3.2.3. Calibration Curve for Microfluidic Detection System

Using the optimal conditions discussed in the previous sections, a standard calibration curve for detection purposes was established by measuring the A_555_/A_666_ ratios for the five Na^+^ control samples with concentrations of 1, 50, 100, 150, and 200 mM, respectively. Five intensity measurements were obtained for each sample, and the average values of the five measurements were used to construct the calibration curve. As illustrated in Figure 5b, the absorbance ratio (Y) showed a logarithmic relationship with the Na^+^ concentration (X), expressed as Y = 0.1054ln(X) + 0.4289, with a correlation coefficient of R^2^ = 0.9989. The high correlation coefficient confirmed the effectiveness and reliability of the proposed Na^+^ detection method. The limit of detection (LOD) of the proposed platform is defined as $LOD=\frac{3\times \sigma }{S}$, where *σ* represents the standard deviation of the blank, and *S* is the slope of the standard deviation of five blank sample readings. Based on this relationship, the LOD of the proposed platform is determined to be 0.7 mM.

### 3.3. Detection Performance of Proposed Microfluidic System for Na^+^ Concentration in Blind Artificial Serum Samples

**Figure 6:**
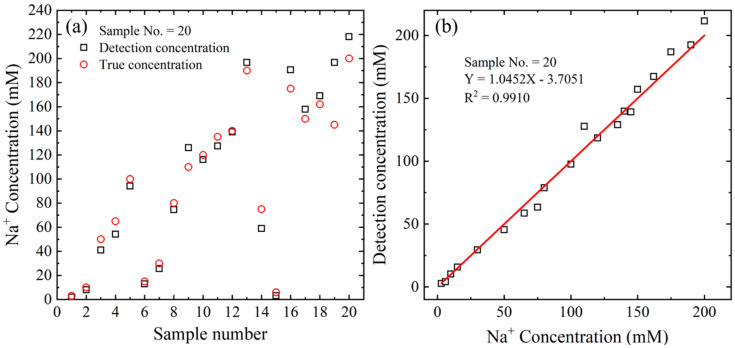
(**a**) Detection results for 20 serum-based Na^+^ samples, and (**b**) correlation between detection results and experimental measurements for 20 serum-based Na^+^ samples.

The practical feasibility of the proposed microfluidic detection system was validated by analyzing 20 artificial serum samples with unknown Na^+^ concentrations in the range of 1–200 mM. Artificial serum samples were prepared by mixing Roche Calibrator for automated systems solution powder with 1 mL of deionized (DI) water to obtain a sodium stock solution with a concentration of 1000 mM, and then diluting the stock solution with DI water for artificial serum blind testing. The detection experiments were conducted under the optimal reaction conditions outlined in Section 3.2, utilizing the calibration equation from Section 3.2.3. The results are presented in Figure 6a,b, with each data point representing the average value from three experiments. The detection and experimental results showed excellent agreement, with a correlation coefficient of R^2^ = 0.9910 (Figure 6b). In addition, the average recovery rate and standard deviation of the blind test were 97.8% and 2.5%, respectively. As a result, the accuracy of the proposed detection platform was validated.

### 3.4. Detection Performance of Proposed Microfluidic System for Na^+^ Concentration in Real-World Serum Samples

**Figure 7:**
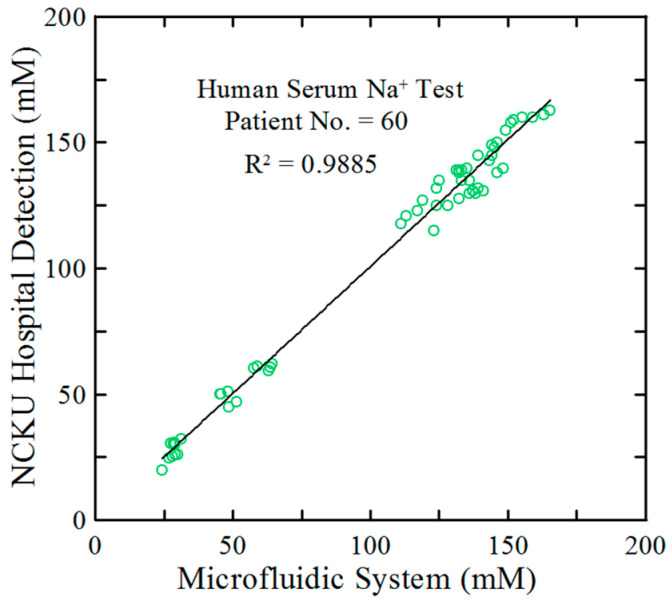
The Na^+^ concentration results obtained from 60 real serum samples were compared between the indirect ISE detection method and the proposed microfluidic system.

Table 2 compares the basic characteristics and performance of the proposed platform with that of several other Na^+^ detection methods reported in the literature. The practical feasibility of the proposed system was further assessed using real serum samples from 60 CKD patients at National Cheng Kung University Hospital (NCKUH) in Taiwan. For comparison, the Na^+^ concentrations in the samples were also measured using the conventional indirect ion-selective electrode (ISE) method (cobas^®^ 8000 modular analyzer, Roche Diagnostics, Switzerland, Basel). In order to evaluate the detection performance of the current rapid microfluidic ion-exchange optode detection system for lower Na^+^ concentrations (i.e., below 100 mM), 40 samples with Na^+^ concentrations ranging from 110 mM to 150 mM were collected from NCKUH. Then, the 20 original samples were taken and each of them were diluted 2.5-fold and 5-fold with deionized water to obtain an additional 40 samples. The results are presented in Figure 7, demonstrating a strong correlation coefficient (R^2^ = 0.9885) between the two sets of results over the considered Na^+^ range. Furthermore, the proposed system exhibits impressive average recoveries and standard deviations of 99.4% and 4.5%, respectively, compared to reference measurements, confirming its suitability.

Despite the promising results and practical advantages of the proposed microfluidic Na^+^ detection system, several key challenges still need to be addressed. First, the current microfluidic Na^+^ detection system relies on serum samples for analysis, requiring whole blood to be preprocessed into serum during the diagnostic process. While microfluidic devices can independently perform this preprocessing, it is rarely integrated into a single microfluidic detection chip. Therefore, future research could focus on developing an integrated microfluidic Na^+^ detection system that incorporates blood sample pretreatment directly onto the detection chip. Second, while the system exhibits high accuracy within the tested concentration range, extreme deviations beyond this range may necessitate additional calibration to ensure measurement accuracy. Moreover, maintaining batch-to-batch consistency in large-scale microfluidic chip production remains a challenge. To overcome this, future efforts will focus on stricter quality control measures and standardized manufacturing processes to enhance reproducibility and scalability.

## 4. Conclusions

This study introduced a microfluidic system composed of a finger-pump detection chip and a micro-spectrometer for measuring Na^+^ concentration in human blood serum. In this system, the serum sample is loaded into the chip and mixed with pre-loaded reagents, driven by two finger-pressure pumps. On completion of the colorimetric reaction, the detection chip is inserted into the detection platform and the absorbance intensity ratio is calculated for wavelengths of 555 nm and 666 nm. Finally, the Na^+^ concentration of the sample is determined from the calculated absorbance ratio using a calibration curve prepared in advance using Na^+^ samples with known concentrations in the range of 1–200 mM.

The microfluidic system showed an excellent linear relationship (R^2^ = 0.9989) between the A_555_/A_666_ ratio and the ion concentrations of five control samples with concentrations of 1, 50, 100, 150, and 200 mM, respectively. Additionally, the system attained an average recovery rate of 97.8% for 20 artificial serum samples, with a strong correlation coefficient of R^2^ = 0.9910. Furthermore, the analysis of 60 real serum samples from CKD patients revealed that, compared to the traditional indirect ISE method, the detection results showed a correlation coefficient of R^2^ = 0.9885, with an average recovery rate and standard deviation of 99.4% and 4.5%, respectively.

Compared to conventional methods, the microfluidic detection system proposed in this study offers numerous advantages, including a faster detection time (less than 3 min), reduced sample consumption (10 μL), improved portability, lower cost, and a straightforward operation. Consequently, it provides a useful tool for determining the Na^+^ concentration in human serum in POC test settings.

## Figures and Tables

**Table 1:** The details of the chemicals.

Model	Vendor	Catalog #	Make
Tris(hydroxymethyl)aminomethane	UNI-ONWARD Corp., New Taipei City, Taiwan	77-86-1	Merck KGaA, Darmstadt, Germany
Hydrochloric acid	UNI-ONWARD Corp., New Taipei City, Taiwan	7647010	Honeywell, Charlotte, NC, USA
SODIUM CHLORIDE	UNI-ONWARD Corp., New Taipei City, Taiwan	7647145	Sigma-Aldrich, St. Louis, MO, USA
Chromoionophore I (ETH 5294)	UNI-ONWARD Corp., New Taipei City, Taiwan	125829-24-5	MedChemExpress, Princeton, NJ, USA
Potassium tetrakis [3,5-bis(trifluoromethyl)phenyl]borate (KTFPB)	UNI-ONWARD Corp., New Taipei City, Taiwan	105560-52-9	Sigma-Aldrich
Sodium ionophore VI	UNI-ONWARD Corp., New Taipei City, Taiwan	80403-59-4	Sigma-Aldrich

**Table 2:** Comparison of analytical methods of sodium ion detection in serum.

Author and Year	Detection Method	Detection Range	Analysis Time	Real-World Samples	Recovery	Ref.
Murugananthan, 2012	Spectroscopy	0–200 mM	6 min	10	98%	[49]
Machini, 2013	Electrochemical	20.1–209 μM	80 s	4	100.2%	[15]
Martinez, 2009	Electrochemical	78.9–349 μM	100 s	5	99.9%	[50]
Chen, 2021	Ion-Selective Electrode	50–200 mM	2 min	-	-	[5]
Yu, 2021	Ion-Selective Electrode	0.05–1000 mM	10 min	16	-	[51]
Current study	Ion Exchange Optodes	1–200 mM	3 min	60	99.4%	-

## Data Availability

The data presented in this study are available upon request from the corresponding author.
